# 

**Published:** 1904-04

**Authors:** 


THE AMERICAN

### Journal of Dental Science.

THIRD SERIES.

THE OLDEST DENTAL MAGAZINE IN THE WORLD.

VOL. XXXV. APRIL, 1904. NO. 4.

EDITOR:
WM. GIRD BEECROFT, D. D. S., Madison, Wis.
ASSOCIATE EDITORS:
JOHN P. BUCKLEY, Ph. G., D. D. S., Chicago.
ALBERT H. KETCHAM, D. D. S., Denver, Colo.
EMORY A. BRYANT, D. D. S., Washington, D. C.
GEO. S MARTIN, D. D. S., L. D. S. Toronto Junction, Ontario.

Publisheu on the 10th day of each month.
Subscription Price, $1.00 per year.
Foreign Countries, $1.50 pe.· year.
Address all communications, both business and editorial to the editor.
**Entered as Second Class Matter, Madison, Wis.**

### NORTHERN OHIO DENTAL ASSOCIATION.

The 45th annual meeting of the Northern Ohio Dental association will be held at Cleveland, Tuesday, Wednesday and Thursday, June 7th, 8th and 9th. The program is a strong one and will be of exceptional interest to the general profession. The motto for the year is the "Annihilation of Pain in Dentistry." Essayists and clinicians have been selected with this thought ever foremost. The best authorities and the most successful men in this line of work will be at this meeting. The members of the profession are cordially invited to attend. It is expected that we will have the largest attendance of any meeting ever held in this section of the country. You can not afford to miss it. Come.—*W. C. Ebersole, Corresponding Secretary.*

